# Hypercontractile esophagus responsive to potassium-competitive acid blockers: a case report

**DOI:** 10.1186/s12876-022-02375-x

**Published:** 2022-06-23

**Authors:** Yuyang Shao, Chen Xie, Huang Feng, Donglin Yan, Weichang Chen

**Affiliations:** Department of Gastroenterology, First Affiliated Hospital of Soochow University, 188 Shizi Road, Suzhou, Jiangsu Province China

**Keywords:** Hypercontractile esophagus, Jackhammer esophagus, High-resolution manometry, Potassium-competitive acid blockers, Case report

## Abstract

**Background:**

Hypercontractile esophagus is a rare hypercontractile esophageal motility disorder. The etiology of hypercontractile esophagus is unknown but an association between acid reflux and hypercontractile esophagus has been suggested. We present the first report on the use of potassium-competitive acid blockers in the treatment of hypercontractile esophagus.

**Case presentation:**

A 43-year-old man presented with dysphagia, chest pain and regurgitation for a period of 1 year. Initial workup showed a twisted lumen with abnormal contractions in the distal esophagus during upper gastrointestinal endoscopy and abnormal acid exposure under 24-h esophageal pH monitoring. The use of standard-dose proton pump inhibitors didn’t relieve his symptoms. Subsequent high-resolution esophageal manometry made a diagnosis of hypercontractile esophagus. Treatment with vonoprazan resulted in symptomatic resolution and abnormal contractions were no longer detected on follow-up high-resolution manometry.

**Conclusions:**

Potassium-competitive acid blockers like vonoprazan offer an alternative therapeutic method for patients with hypercontractile esophagus who are refractory to proton pump inhibitor therapy. The use of potassium-competitive acid blockers in hypercontractile esophagus warrants further research and may provide evidence for an acid-related etiology of hypercontractile esophagus.

## Background

Hypercontractile esophagus (HE), also known as jackhammer esophagus, is characterized by multipeaked peristaltic contractions of the esophagus. Patients can present with dysphagia, noncardiac chest pain, heart burn and regurgitation. The Chicago Classification version 4.0 describes the diagnostic criteria for HE as at least 20% swallows with a distal contractile integral (DCI) of > 8000 mmHg.s.cm on high-resolution manometry (HRM) [1]. HE is a rare disease and its pathophysiology is complex. Some reports suggested a link between HE and gastroesophageal reflux disease (GERD), but empirical proton pump inhibitor (PPI) treatment did not result in clinical remission in some cohorts [2–4]. Potassium-competitive acid blockers (P-CAB) are a new therapeutic strategy in acid-related diseases. Here, we report a case of HE whose symptoms were improved by P-CAB therapy.

## Case presentation

**Fig. 1:**
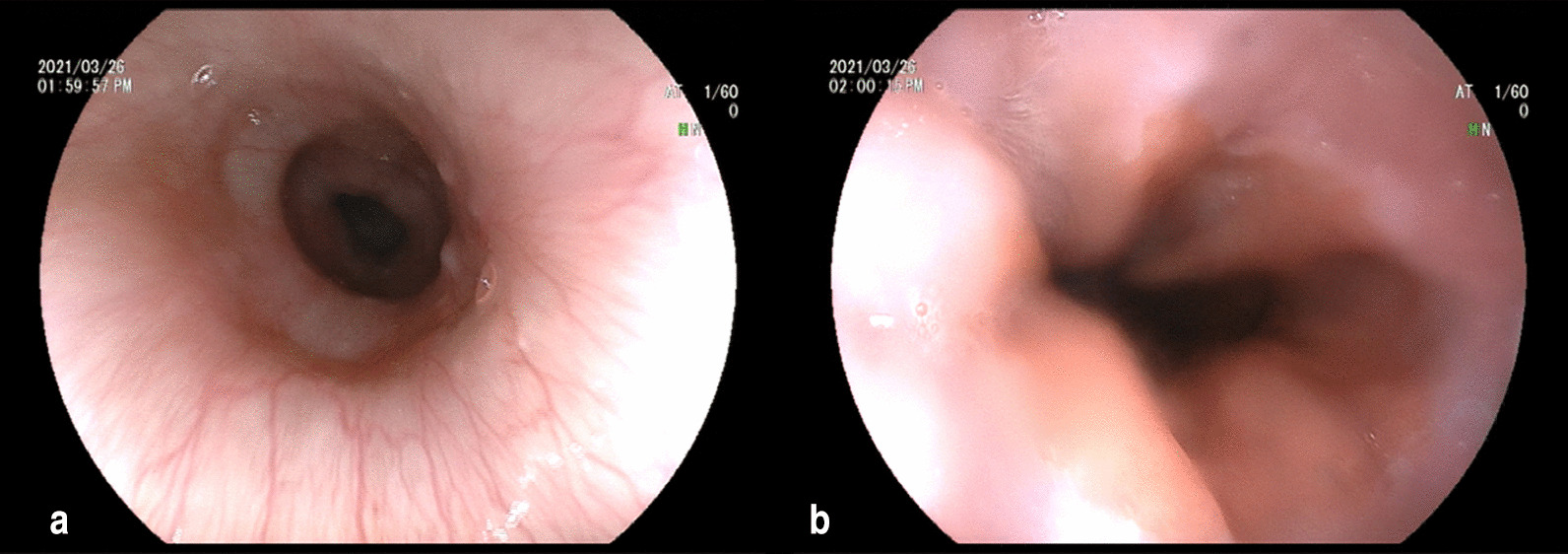
**a** Upper gastrointestinal endoscopy revealed abnormal contractions in the distal esophagus; **b** and loose adherence of the esophagogastric junction without signs of esophagitis

**Fig. 2:**
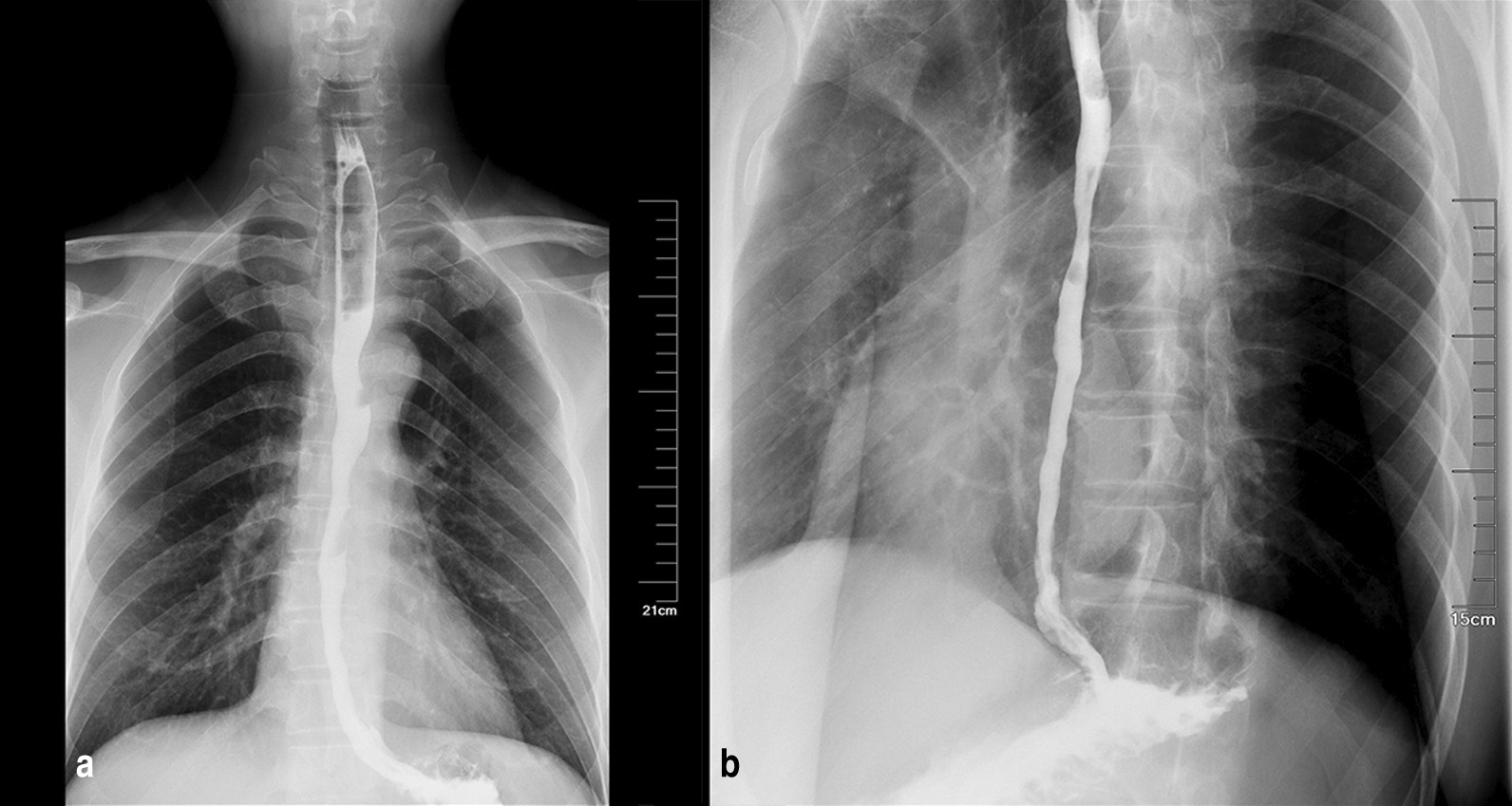
Barium esophagogram showed irregular mucosa changes along the distal esophagus on an anterior–posterior chest X-ray (**a**) and a lateral chest X-ray (**b**)

**Fig. 3:**
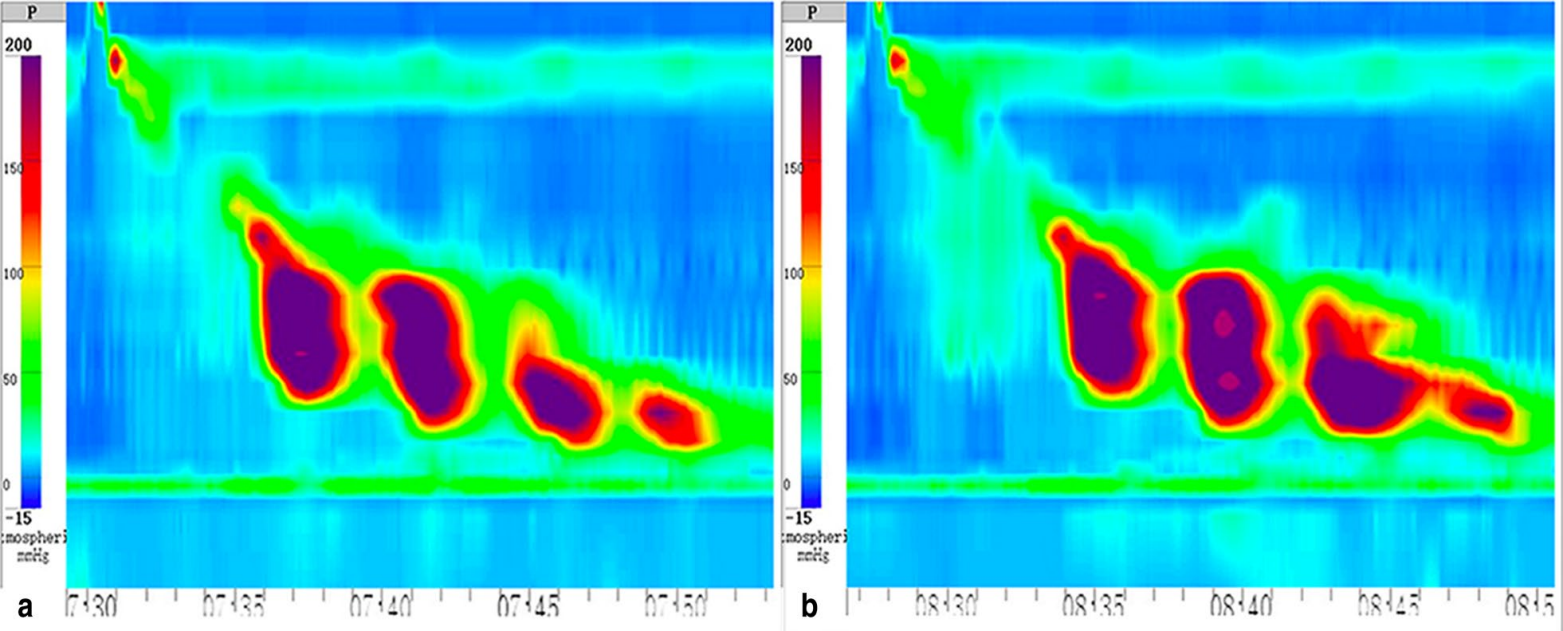
HRM of the patient with HE. The median IRP was 24.6 mmHg and distal latency (DL) was 10.4 s. **a** This hypercontractile swallow has a DCI of 16,819 mmHg.s.cm. **b** This hypercontractile swallow has a DCI of 20,656 mmHg.s.cm

A 43-year-old male was referred to our hospital with progressive dysphagia and chest pain for nearly one year. He also had frequent instances of regurgitation of food especially when lying down at night. He denied any history of smoking, alcohol drinking or cardiac problems. Physical examination and basic laboratory tests were normal. An electrocardiogram did not show any abnormal findings. A computerized tomography (CT) scan of the chest showed no neoplastic or vascular obstruction outside the esophagus. Upper gastrointestinal endoscopy showed abnormal contractions in the distal esophagus with grossly normal-appearing mucosa (Fig. 1a) and loose adherence of the esophagogastric junction without signs of esophagitis (Fig. 1b). There was no eosinophilic infiltration on biopsy. Barium esophagogram showed irregular mucosa changes along the distal esophagus (Fig. 2). 24-h esophageal pH monitoring showed abnormal acid exposure with a DeMeester score of 15.24. The patient was diagnosed as non-erosive reflux disease and received PPI therapy (Esomeprazole, 20 mg, QD) for 12 weeks without symptom improvement. Subsequent HRM revealed intermittent hypertensive esophageal contractions with 7 of 10 swallows with a DCI > 8000 mmHg.s.cm and a maximum DCI value of 20,656 mmHg.s.cm. The median integrated relaxation pressure (IRP) was high at 24.6 mmHg (Fig. 3). HE was diagnosed based on the HRM study.

**Fig. 4:**
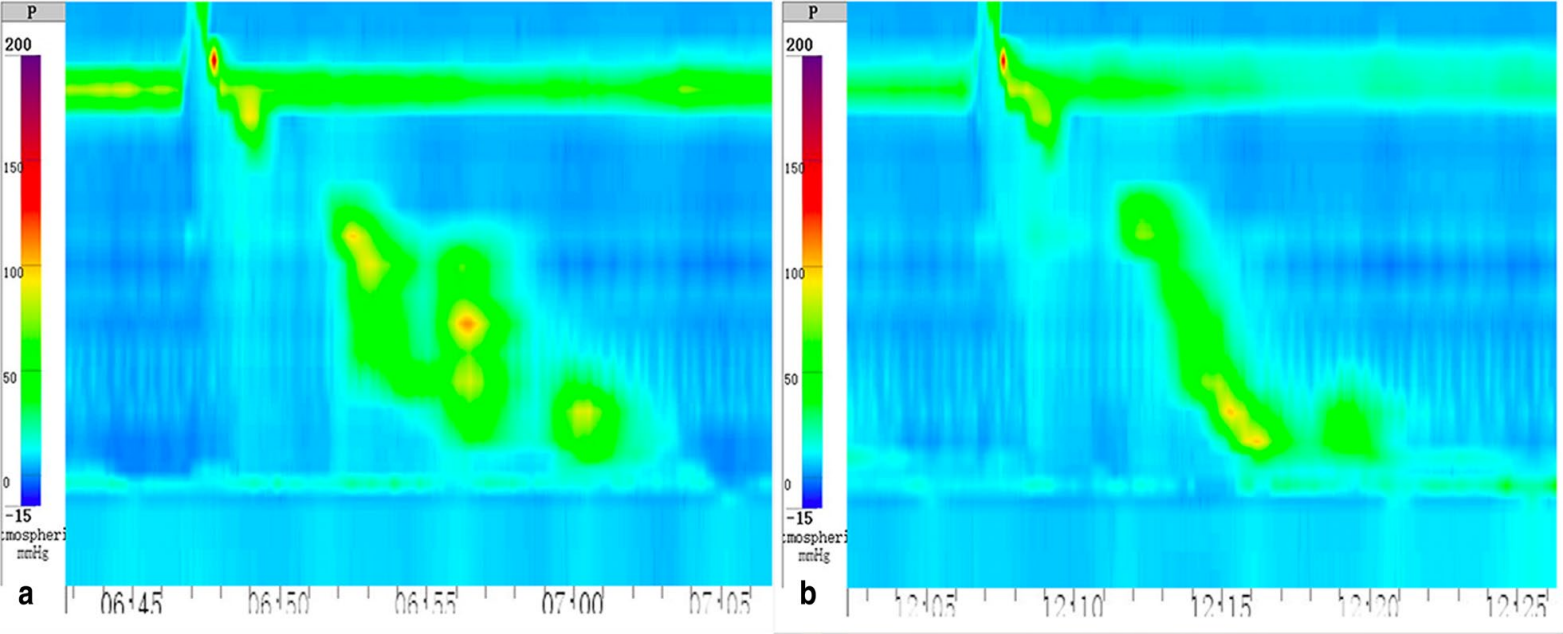
HRM of the patient after 14 weeks of treatment with P-CAB. The parameters are within normal limits with a median IRP of 2.3 mmHg and a mean DCI of 2173 mmHg.s.cm. **a** This swallow has a DCI of 2275 mmHg.s.cm. **b** This swallow has a DCI of 1224 mmHg.s.cm

The patient was treated with P-CAB (Vonoprazan, 20 mg, QD) for 4 weeks with markedly symptoms improved. At follow-up 14 weeks later, the patient felt well with much improved symptoms with no regurgitation and complete resolution of his chest pain. He then suspended the use of vonoprazan for one week and no signs of recurrence were detected. After discontinuation of vonoprazan for three weeks, the follow-up HRM demonstrated a mean DCI of 2173 mm Hg.s.cm and a median IRP of 2.3 mmHg (Fig. 4). 24-h esophageal pH monitoring showed a decreased acid reflux with a DeMeester score of 5.22. He refused to undergo barium esophagogram again as the symptoms had alleviated a lot.

## Discussion and conclusions

HE is a rare motility disorder associated with dysphagia, noncardiac chest pain, regurgitation, and heartburn. A full diagnostic evaluation into other etiologies is necessary prior to the diagnosis of HE. It includes upper endoscopy with biopsies and barium esophagogram to access for esophagitis, stenosis and achalasia, 24-h pH monitoring to rule out gastroesophageal reflux, endoscopic ultrasonography (EUS) and CT scan to exclude the neoplasm in or out of esophageal wall. A proportion of patients with normal investigations should be considered for esophageal studies like esophageal HRM as they may reveal an underlying dysmotility disorder. HRM is the gold standard technique to diagnose HE based on the recognition of hypercontractility quantified by DCI.

The pathophysiology of HE is complex with varying contributors including gastroesophageal acid reflux, hiatal hernia, eosinophilic esophagitis, excessive excitation of vagal nerve, spinal cord injury, intake of opioids and tumors [5–7]. Early acid perfusion studies have demonstrated the ability of acid to provoke esophageal spasms, motility changes and the perception of non-cardiac chest pain [8], suggesting that gastroesophageal acid reflux is highly involved in the pathogenesis of HE. The presence of esophageal hypersensitivity in patients may account for the failure of PPI treatment due to increased reflux to the proximal esophagus [9, 10]. It can be speculated that patients with HE present with visceral hypersensitivity that increases esophageal perception to low-intensity stimuli, leading to a hypercontractile state of the esophagus [11, 12]. As in this case, the patient was nonresponsive to PPI therapy but treatment with vonoprazan relieved his symptoms near complete resolution. Vonoprazan is a novel acid inhibitor and classified as a P-CAB. It competitively blocks the potassium-binding site of H + /K + -adenosine triphosphatase(ATPase) during gastric acid secretion in gastric parietal cells [13]. It is characterized by rapid, stable and long-lasting effects compared with conventional PPIs and is specially developed for the treatment of acid-related gastrointestinal diseases such as GERD. In view of the fact that gastroesophageal reflux may be a cause or result of HE, P-CAB therapy can be considered as an alternative approach for HE patients with GERD overlap when PPI therapy fails. However, the duration and efficacy of P-CAB therapy in HE needs further evaluation and requires longer follow-up.

As there have not been any prospective studies addressing the treatment of HE, its management approach is yet to be validated. Pharmacotherapy including calcium channel blockers, nitrates, phosphodiesterase-5 inhibitors, anticholinergics, and low dose antidepressants is effective in a subset of patients with HE, but lacks long-term follow-ups to assess the efficacy of these drugs [14, 15]. Interventional procedures such as botulinum toxin injection, pneumatic dilation and per-oral endoscopic myotomy (POEM) show favorable results when patients are refractory to medications [5, 16, 17].

In summary, this report provides the first insight into the use of P-CAB therapy in patients with HE, which strengthens the link of causality between acid reflux and HE. Given the low incidence of HE, a multicenter randomized trial is required to obtain an evidence-based answer to whether the use of P-CAB is warranted in HE.

## Data Availability

The data used to support the findings of this case report are included within the article.

## Abbreviations

HE: Hypercontractile esophagus
HRM: High-resolution manometry
PPI: Proton pump inhibitor
P-CAB: Potassium-competitive acid blockers
GERD: Gastroesophageal reflux disease
IRP: Integrated relaxation pressure
DCI: Distal contractile integral
DL: Distal latency
CT: Computerized tomography
EUS: Endoscopic ultrasonography
POEM: Per-oral endoscopic myotomy
ATPase: Adenosine triphosphatase

## References

[1] Yadlapati R, Kahrilas PJ, Fox MR, et al. Esophageal motility disorders on high-resolution manometry: Chicago classification version 4.0©. *Neurogastroenterol Motil*. 2021;33(1):e14058. doi:10.1111/nmo.14058 10.1111/nmo.14058 PMC8034247 33373111

[2] Mallet AL, Ropert A, Bouguen G (2016). Prevalence and characteristics of acid gastro-oesophageal reflux disease in Jackhammer oesophagus. Dig Liver Dis.

[3] Kristo I, Schwameis K, Maschke S (2018). Phenotypes of Jackhammer esophagus in patients with typical symptoms of gastroesophageal reflux disease responsive to proton pump inhibitors. Sci Rep.

[4] Borjesson M, Rolny P, Mannheimer C, Pilhall M (2003). Nutcracker oesophagus: a double-blind, placebo-controlled, cross-over study of the effects of lansoprazole: acid suppression for nutcracker oesophagus. Aliment Pharmacol Ther.

[5] Tanaka S, Toyonaga T, Kawara F (2018). A case of Jackhammer esophagus caused by eosinophilic esophagitis in which per-oral endoscopic myotomy resulted in symptom improvement. Clin J Gastroenterol.

[6] Clément M, Zhu WJ, Neshkova E, Bouin M (2019). Jackhammer esophagus: from manometric diagnosis to clinical presentation. Can J Gastroenterol Hepatol.

[7] Milito P, Siboni S, Bonavina L. High-Pressure Tactics: Jackhammer Esophagus—Diagnosing Is Easier than Treating. *Dig Dis Sci*. Published online October 21, 2021. doi:10.1007/s10620-021-07279-6 10.1007/s10620-021-07279-6 PMC8529866 34674073

[8] Crozier RE, Veerman JM. Acid-provoked esophageal spasm as a cause of noncardiac chest pain. 1991;86(11):6. 1951232

[9] Abdallah J, George N, Yamasaki T, Ganocy S, Fass R (2019). Most patients with gastroesophageal reflux disease who failed proton pump inhibitor therapy also have functional esophageal disorders. Clin Gastroenterol Hepatol.

[10] Rohof WO, Bennink RJ, de Jonge H, Boeckxstaens GE (2014). Increased proximal reflux in a hypersensitive esophagus might explain symptoms resistant to proton pump inhibitors in patients with gastroesophageal reflux disease. Clin Gastroenterol Hepatol.

[11] Yamasaki T, Fass R (2017). Reflux hypersensitivity: a new functional esophageal disorder. J Neurogastroenterol Motil.

[12] Fass R (2009). Proton pump inhibitor failure—what are the therapeutic options?. Am J Gastroenterol.

[13] Sugano K. Vonoprazan fumarate, a novel potassium-competitive acid blocker, in the management of gastroesophageal reflux disease: safety and clinical evidence to date. *Therap Adv Gastroenterol*. 2018;11:1756283X17745776. doi:10.1177/1756283X17745776 10.1177/1756283X17745776 PMC5784563 29383028

[14] Sirinawasatien A, Sakulthongthawin P (2021). Manometrically jackhammer esophagus with fluoroscopically/endoscopically distal esophageal spasm: a case report. BMC Gastroenterol.

[15] Li JY, Zhang WH, Huang CL, Huang D, Zuo GW, Liang LX (2017). Deanxit relieves symptoms in a patient with jackhammer esophagus: a case report. World J Gastrointest Endosc.

[16] Marjoux S, Brochard C, Roman S (2015). Botulinum toxin injection for hypercontractile or spastic esophageal motility disorders: may high-resolution manometry help to select cases?: HRM and esophageal botulinum toxin. Dis Esophagus.

[17] Inoue H, Sato H, Ikeda H (2015). Per-oral endoscopic myotomy: a series of 500 patients. J Am Coll Surg.

